# Toxicity and Affecting Factors of *Bacillus thuringiensis* var. *Israelensis* on *Chironomus kiiensis* Larvae

**DOI:** 10.1673/031.012.12601

**Published:** 2012-11-03

**Authors:** Chuan-Wang Cao, Li-Li Sun, Rong-Rong Wen, Xiao-Peng Li, Hong-Qu Wu, Zhi-Ying Wang

**Affiliations:** ^1^ Department of Forest Protection, Northeast Forestry University, Harbin 150040, China; ^2^ Hubei Academy of Agricultural Sciences, Wuhan 430064, China

**Keywords:** bioassay

## Abstract

*Bacillus thuringiensis* var. *israelensis* (Bti) is a suitable agent for controlling *Chironomus kiiensis*, a major pest polluting water. In this study, laboratory bioassays were used to study toxicity and affecting factors of Bti on *C. kiiensis* larvae. Tests were conducted using three commercial Bti formulations (oil miscible suspension, 1,200 ITU/mL; wettable power, 1,200 ITU/mg; technical material, 5,000 ITU/mg) of Bti. The toxicity of Bti formulations to third and fourth instar *C. kiiensis* larvae was in decreasing order of technical material, oil miscible suspension, and wettable powder, based on the 12 and 24 hour LC_50_ values. Increasing larval densities (from 10 to 30 per bioassay cup) increased the LC_50_ values for fourth instar *C. kiiensis* larvae. The LC_50_ values for fourth instar larvae reared in sand substrate were higher than those from soil substrate, and autoclaved substrates significantly increased the LC_50_ values. The technical material of Bti at 12 and 24 hours responded similarly to changes in temperature between 30° C and 15° C, but the LC_50_ values at a range of tested temperatures showed distinct differences in time points.

## Introduction

Chironomidae (Diptera) is a species-rich family of flies, with almost 15,000 species described worldwide (Cranston 1994). As an important component of the aquatic insect community, chironomids have proven to be useful as biological indicators because of their sensitivity to chemical changes in aquatic ecosystems (Dudley and Blair 1992). In recent years, chironomid larvae have infested municipal water supply systems in more than 10 Chinese provinces (Zhang et al. 2004). Adult emergence of chironomid midges can occur at nuisance levels in areas surrounding urban and suburban aquatic habitats (Ali 1996). At a shallow wetland in northeast Florida, USA, chironomid populations have existed for several months each year at nuisance levels that interfere with human activities, necessitating control measures (Ali et al. 2008). *Chironomus kiiensis* Tokunaga has been reported to be the most prevalent chironomid species in Korea out of about 50 species recorded to date (Ree 1993). In China, *C. kiiensis* is mainly distributed in city water bodies in the southern part of the country, affecting water quality and being a potential disease vector (Lei et al. 2004). Since 2002, the biological mosquito larvicide *Bacillus thuringiensis* var. *israelensis* (Bti) has been used to control these insects. Lei et al. (2005) investigated the toxicity characteristics of Bti IPS82 on *C. kiiensis* and reported that it would be feasible to use Bti IPS82 to control *C. kiiensis* in city water sources. In this study, we describe the toxicity and affecting factors of three formulations of Bti on larvae of *C. kiiensis* to further improve biological control strategies.

## Materials and Methods

### Test materials

Oil miscible suspension (1,200 ITU/mL), wettable power (1,200 ITU/mg), and technical material (5,000 ITU/mg) of Bti were obtained from Hubei Kangxin Agro-industry Co., Ltd. (http://www.btrdc.com/).

### *C. kiiensis* larvae

*C. kiiensis* egg masses were collected from water bodies in Shenzhen city, China, and were cultured in accordance with standard protocols (EPA 1993). Briefly, instead of collecting and separating eggs masses, the midges were reared in mixed-age cultures. Instar determination was achieved using length estimates. Larval midges were harvested directly from the mixed-age cultures and used in the bioassays.

### Bioassay

The suspension stocks were prepared by putting 100 mg Bti into 1 L deionized water and shaking at 200 rpm for 30 minutes. Suspensions for use in bioassays were prepared by serial dilution of Bti in dechlorinated water. The acute toxicity of each compound was evaluated individually using 12 and 24 hour static toxicity tests. Ten larvae were randomly assigned to each of three replicates per treatment, giving a total of 30 larvae in each of the control and seven test treatments (0.01, 0.05, 0.1, 0.5, 1, 5, and 10 mg/L). Tests were conducted in 100-mL plastic cups containing 50 mL of test solution, which was comprised of dechlorinated water. Test vessels were not aerated and test solutions were not renewed for 24 hours. The test vessels were covered with clear plastic film to minimize evaporation. Tests were conducted at room temperature with a photoperiod of 16:8 L:D. The number of immobilized midges was recorded at 12 and 24 hours using a dissecting microscope (× 10 magnification). Immobilization was defined as the cessation of all visible signs of movement or activity when viewed under the dissecting microscope. Each experiment was repeated using a different batch of midge larvae. An experiment was considered valid if mortality in the control did not exceed 10% at the end of the test and dissolved oxygen did not fall below 20% saturation. Bioassays evaluating the effects of larval age were conducted by measuring the toxicity of oil miscible suspension (1,200 ITU/mL), wettable powder (1,200 ITU/mg), and technical material (5,000 ITU/mg) of Bti on third and fourth instar *C. kiiensis* larvae. River sand and wetland soil were used to study the effects of substrate on the toxicity of Bti in this study. River sand was washed for 20 minutes in distilled water, oven dried, and passed through a 74-µm mesh sieve onto a stainless steel tray. Topsoil was collected from Zhalong wetland, Heilongjiang, China. The basic physical and chemical characteristics of soil are shown in Table 1. Bioassay data were pooled, and a probit analysis was conducted using POLO probit analysis software. Significant differences in the resistance levels of the insects were based on non-overlapping 95% confidence intervals (Tian et al. 2011).

## Results

### Toxicity of Bti on fourth instar *C. kiiensis* larvae

The larvicidal activity of three Bti preparations is summarized in Table 2. The third and fourth instar *C. kiiensis* larvae were susceptible to wettable powder, oil miscible suspension, and technical material of Bti. The 12 and 24 hour LC_50_S of the three Bti formulations showed similar changes for third and fourth instar *C. kiiensis* larvae and were in decreasing order of wettable powder, oil miscible suspension, and technicial material. The young third instar larvae were more susceptible than fourth instar larvae.

### Influence of population density on the toxicity of Bti on *C. kiiensis* larvae

Table 3 shows the effect of population density on the toxicity of Bti by testing the 12 and 24 hour LC_50_ of technical material on fourth instar larvae. The 12 and 24 hour LC_50_S of Bti with a density of 30 larvae/cup were 2.40 and 2.08 fold higher than at a density of 10 larvae/cup, respectively. The results showed that Bti activity in low densities was higher than in greater densities.

### Influence of substrate on toxicity of Bti on *C. kiiensis* larvae

Table 4 depicts the influence of soil, autoclaved soil, sand, and autoclaved sand substrates on the toxicity of Bti on fourth instar *C. kiiensis* larvae. The 12 hour LC_50_S of Bti on fourth instar *C. kiiensis* larvae residing in autoclaved soil, sand, and autoclaved sand were increased by 10.43, 9.50 and 15.39 fold compared to 10 larvae per cup in soil, respectively. Moreover, the 24 hour LC_50_S of the fourth instar *C. kiiensis* larvae in autoclaved soil, sand, and autoclaved sand were also significantly higher than in soil substrate. However, the autoclaved substrates significantly influenced the toxicity of Bti on the *C. kiiensis* larvae. After 12 hours, the LC_50_S for larvae from autoclaved soil and autoclaved sand were 10.43 and 1.62 fold higher than those from soil and sand, respectively.

### Influence of temperature on the toxicity of Bti on fourth instar *C. kiiensis* larvae

To investigate the influence of temperature on the toxicity of Bti, five temperatures were selected to measure LC_50_ values for technical material of Bti bioassayed against fourth instar *C. kiiensis* larvae (10 larvae per cup, sand substrate) at a range of constant temperatures. The results showed that Bti at two time points responded similarly to changes in temperature between 30° C and 15° C. Between 30° C and 27° C, the Bti at 12 hours showed a steep (3.60 fold) decline in activity, whereas the corresponding decline in activity at 24 hours was only 1.02 fold. The 12 hour LC_50_ values at the tested temperature extremes for Bti were 3.11 mg/L (30° C) and 25.51 mg/L (15° C), while 24 hour LC_50_ values were 0.41 mg/L (30° C) and 2.25 mg/L (15° C). The LC_50_ values at a range of tested temperatures showed a distinct difference in time points, such as the 12 hour LC_50_ being 23.49 fold higher than the 24 hour LC_50_.

### Discussion

Since Bti was initially described and applied to control mosquito larvae (Goldberg and Margalit 1977), Bti-based products have been used to control many mosquito (Goldberg and Margalit 1977; Margalit and Dean 1985; Amalraj et al. 2000; Fillinger et al. 2003) and chironomid species (Lei et al. 2004, 2005; Ali et al. 2008) worldwide. To date, some effective commercial Bti products have been registered for dipteran insect control (Russell and Kay 2008). For example, VectoBac WDG (3000 ITU/mg) has been recorded to be effective against *Chironomus tepperi* (Stevens et al. 2004). Formulations of Bti used at an appropriate dosage for mosquito control do not have a significant impact on most other animals or plants (Boisvert and Boisvert 2000). The chironomid species *C. kiiensis* is susceptible to three Bti formulations, with LC_50_ values (24 hours) ranging from 0.13 to 0.37 mg/L for third instar larvae, and from 0.32 to 1.57 mg/L for fourth instar larvae. These results were similar to mosquitoes (Nayar et al. 1999) and other chironomids (Charbonneau et al. 1994), with the susceptibility of older instar larvae to Bti declining as their age increases. Larval density can significantly affect the toxicity of Bti on mosquito larvae and chironomids (Nayar et al. 1999; Charbonneau et al. 2004). Stevens et al. (2004) also reported that increasing larval densities from 10 to 30 per bioassay cup resulted in increased LC_50_ values for both age groups, significantly so in the case of older larvae (higher density LC_50_ 0.80 mg/L). Our results showed a similar variation, in which the 12 and 24 hour LC_50_S of Bti at a density of 30 larvae/cup were 2.40 and 2.08 fold higher than at a density of 10 larvae/cup, respectively. Additionally, substrate type and temperature also affected Bti efficacy. Our data demonstrated that the LC_50_ values (fourth instar larvae) using sand substrate were higher than those in soil substrate. The characteristics of soil inhabited by chironomid larvae may therefore affect the Bti activity. Charbonneau et al. (1994) demonstrated that sediment characteristics might have an effect on the response of chironomids to Bti. Autoclaved substrates significantly increased the LC_50_ values, thus suggesting that an increased toxicity in treated substrates might be due to the contribution of other microorganisms in original substrates. However, Stevens et al. (2004) found soil substrate reduced Bti efficacy relative to sand, and autoclaved soil substrate did not significantly affect the toxicity. These authors presumed that Bti toxins might be absorbed by clay soil particles and thus become unavailable to chironomid larvae. Previous studies observed that Cry proteins of Bt were absorbed by soil particles and became unavailable to ingestion by microbes (Venkateswerlu et al. 1992; Tapp et al. 1994; Koskella et al. 1997; Tapp et al. 1998; Pagel-Wieder et al. 2007). Therefore, further studies should investigate possibilities of Bt toxin protein biodegradation, and how to maintain an efficient soil to target insects.

Temperature is also a major factor affecting Bti toxicity (Charbonneau et al. 1994; Nayar et al. 1999). The toxicity of VectoBac WDG and a Bti spore/crystal mixture to *C. tepperi* has been shown to display large variations in LC_50_ values at 15° C. In this study, the data with technical materials of Bti and *C. kiiensis* larvae showed similar changes at temperatures between 30° C and 15° C. The Bti toxins can produce toxicity in the insect midgut after ingestion (Cao et al. 2010), and it is likely that an increased larval feeding activity at higher temperatures contributes substantially to the observed increases in Bti toxicity (Stevens et al. 2004). To effectively control the pestiferous chironomid larvae, it is prerequisite to optimize field conditions for maximizing the toxicity of Bti.

**Table 1.**
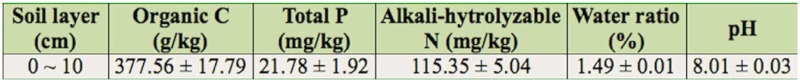
Physical and chemical properties of soil in Zhalong wetlands.

**Table 2.**
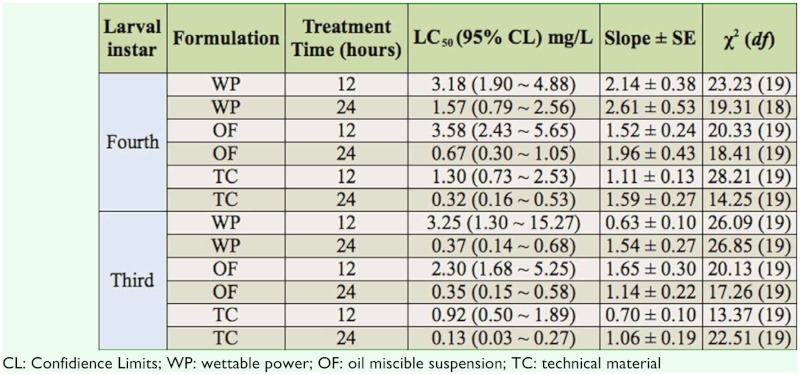
The toxicity of *Bacillus thuringiensis* var. *israelensis* to *Chironomus kiiensis* larvae.

**Table 3.**
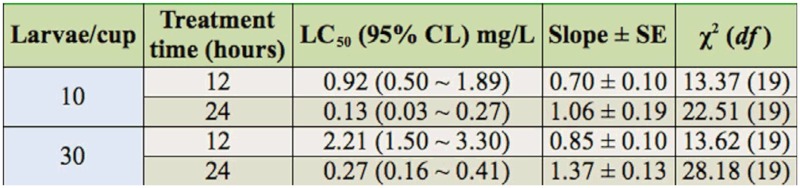
Influence of population density on the toxicity of *Bacillus thuringiensis* var. *israelensis* to fourth instar *Chironomus kiiensis* larvae. The chemical used was technical material.

**Table 4.**
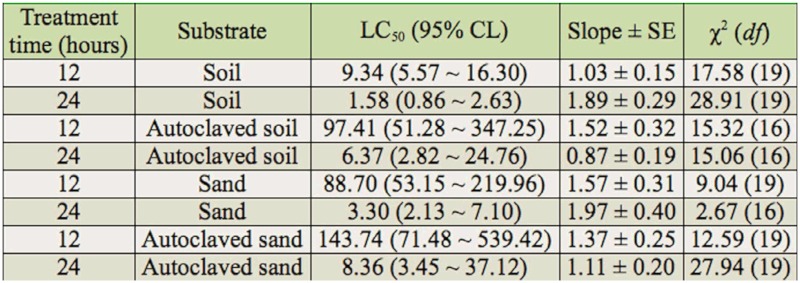
Influence of substrate on the toxicity of *Bacillus thuringiensis* var. *israelensis* to fourth instar *Chironomus kiiensis* larvae at ten larvae per cup.

**Figure 1.**
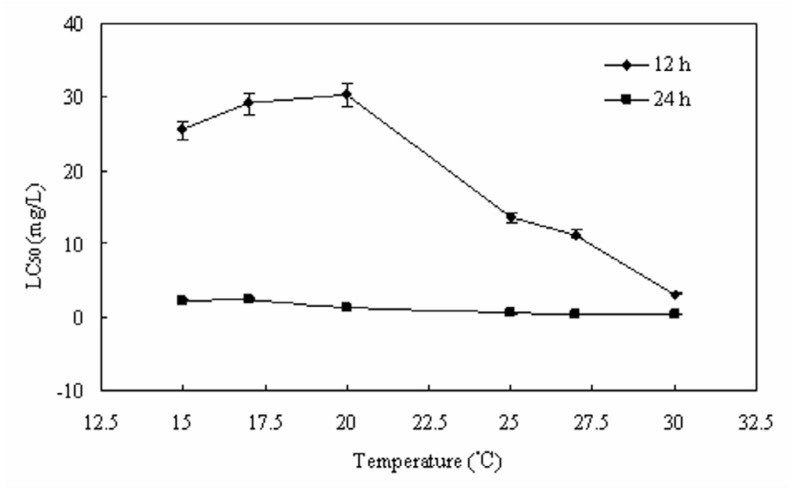
LC_50_ values for technical materials of *Bacillus thuringiensis* var. *israelensis* bioassayed against fourth instar *Chironomus kiiensis* larvae ( 10 larvae per cup, sand substrate) at a range of constant temperatures. Error bars represent 95% confidence limits. High quality images are available online.

## Abbreviations

Bti,: *Bacillus thuringiensis* var. *israelensis*
